# Efficacy of dietary *Ceratonia silique* and *Zingiber offcinale* on the immune-antioxidant-signaling pathways, growth, physiological response, and ammonia resistance in *Oreochromis niloticus* reared under unchanged water

**DOI:** 10.1007/s10695-025-01496-w

**Published:** 2025-05-22

**Authors:** Mohamed F. A. Abdel-Aziz, Mona S. Azab, Ahmed R. Mohamed, Ashraf Y. El-Dakar, Dalia S. Hamza, Gehad E. Elshopakey, Ahmed Shehab, Afaf N. Abdel Rahman

**Affiliations:** 1 Department of Aquaculture and Biotechnology, Faculty of Aquaculture and Marine Fisheries, Arish University, Arish, Egypt; 2 Biology Department, College of Science, Jouf University, 72341 Sakaka, Saudi Arabia; 3 Department of Zoology, Faculty of Science, Benha University, Benha, 13518 Egypt; 4 Department of Clinical Pathology, Faculty of Veterinary Medicine, Mansoura University, Mansoura, 35516 Egypt; 5 Department of Veterinary Diseases, Faculty of Veterinary Medicine, Delta University for Science and Technology, Gamasa, 35712 Egypt; 6 Department of Nutrition and Clinical Nutrition, Faculty of Veterinary Medicine, Benha University, Benha, Egypt; 7 Department of Aquatic Animal Medicine, Faculty of Veterinary Medicine, Zagazig University, PO Box 44511, Zagazig, Egypt

**Keywords:** Gene expression, Herbal additives, Immune response, Nile tilapia, Zero water exchange

## Abstract

Prioritizing water management and maintaining its quality for as long as possible, while lowering related stressors, are crucial for sustainable aquaculture. To achieve this equilibrium, enriched aquafeed with natural immunostimulants is essential to success. In this trend, 6 weeks feeding trial was conducted to evaluate the effects of *Ceratonia siliqua* syrup (CSS) and *Zingiber officinale* powder (ZOP) in Nile tilapia (*Oreochromis niloticus*) reared under a zero-water exchange. The immune-antioxidant, growth, physiological responses, and the antioxidant/inflammatory pathways-associated genes as well as ammonia tolerance were evaluated. Fish (weighing 25.85 ± 1.42 g) were randomly housed into six groups (*n* = 30 fish/group; ten fish/replicate; three replicates/group). The control group was fed a basal diet without any additives. The second (CSS1.25) group was fed a diet supplemented with 1.25% CSS. The third (ZOP0.5) and fourth (ZOP1) groups were fed diets supplemented with 0.5 and 1% ZOP. The fifth (CSS1.25 + ZOP0.5) and sixth (CSS1.25 + ZOP1) groups were fed diets supplemented with 1.25% CSS and 0.5 or 1% ZOP. All treatments were kept without water exchange for 6 weeks. Findings revealed the most notable improvement (*P* < 0.05) in growth rate (final body weight and specific growth rate) and survival rate in fish fed with dietary ZOP and CSS1.25 + ZOP0.5 diets. Hepato-renal markers (alanine and aspartate aminotransferases, urea, glucose, and cortisol) and lipid peroxides (malonaldehyde) were declined by CSS and/or ZOP diets. Immuno-antioxidants (immunoglobulin M, lysozyme, superoxide dismutase (SOD), and reduced glutathione) were significantly boosted (*P* < 0.05) in the ZOP1 and CSS1.25 + ZOP0.5 groups. In addition, CSS and/or ZOP diets markedly (*P* < 0.05) upregulated antioxidant-linked genes (*SOD* and glutathione peroxidase) and downregulated the stress gene (heat shock protein 70) and pro-inflammatory cytokines (interleukin-1β and tumor necrosis factor-alpha). In addition, CSS and/or ZOP diets decreased fish mortality during ammonia stress. The ZOP1 diet was significantly reported of having the best outcomes (*P* < 0.05) throughout the measured indices. Overall, our findings demonstrate that dietary ZOP and CSS at the optimum doses can improve growth, immune response, and physiological functions of *O. niloticus* reared in stressful conditions (unchanged water) for the sustainable aquaculture industry.

## Introduction

Aquaculture is an indispensable enterprise for satisfying the growing needs for animal protein, since fish are a crucial provider of high-quality proteins for human nourishment (FAO 2023; Munguti et al. 2024). One of the foremost frequently grown fish species is Nile tilapia (*Oreochromis niloticus*), which is acclaimed for its large market demand, quick growth, and adaptability (El-Sayed and Fitzsimmons 2023). Increasing feed efficiency, controlling pathogens, stocking fish at a high pace, and maintaining outstanding water quality for as long as feasible are all necessary to achieve the optimum economic return and sustainable aquaculture (Boyd et al. 2020; Kaleem and Bio Singou Sabi 2021). As a consequence of the high stress levels caused by high stocking and the ensuing inadequate water quality, tilapias propagated in intensive output systems may be more predisposed to diseases and immune suppression, which diminishes fish productivity (Fatima et al. 2021; Komal et al. 2024). Hence, it is crucial to create strategies for lessening the negative impacts of stress (Ma et al. 2023).

Currently, a lot of attention is directed to diverse feed supplements in aquafeeds because of their nutritional potential as well as their ability to support health (Ma and Hu 2024). Usually, functional feeds are given to aquatic species to maintain their health under various production environments (Abdel Rahman et al. 2018, 2023; Van Doan et al. 2023). Accordingly, medicinal herbs and their extracts as members of feed additives in aquafeeds could augment growth, overall health, and resistance to stressors (Ayyat et al. 2022). Since, one of the major obstacles in aquaculture is to raise fish immunity, which is a fundamental defense strategy against infections, medicinal plants can also exert an immunostimulant characteristic (Harikrishnan et al. 2011). This is achieved by their bioactive constituents through the activation of non-specific and specific immune responses, thus, boosting the stressors and disease-resistance potentials of aquatic organisms (Harikrishnan et al. 2010; Ahmadifar et al. 2021a). These compounds included alkaloids, essential oils, flavonoids, glycosides, lectins, polyphenolics, polypeptides, polysaccharides, saponins, tannins, and terpenoids (Ahmadifar et al. 2021b; Mbokane and Moyo 2022). Many fishes’ immunological characteristics, such as phagocytic, lysozyme, respiratory burst, peroxide, complement, and anti-protease activities, as well as immune-related cytokines, have been improved when medicinal herbs are applied (Mehrinakhi et al. 2021; Sadeghi et al. 2021; Abdel Rahman et al. 2025a, b; Ibrahim et al. 2025). Correspondingly, they have the potential to substitute chemotherapies and minimize economic losses (Karataş 2024).

In this regard, a perennial evergreen tree and member of the Fabaceae family is carob (*Ceratonia siliqua* L.). This species is an economically and environmentally significant plant and grows in nations with Mediterranean climates (Battle and Tous 1997; Pazır and Yuksel 2016). Its fruit can be directly consumed or frequently applied as syrup and flour. A common product, carob syrup has considerable amounts of phenolic compounds, sugar, and minerals. Carob has antitoxic, antiseptic, antimicrobial, cholesterol-, lipid-, and glucose-lowering qualities (Kaderi et al. 2015; Kahkahi et al. 2024). Another noteworthy medicinal perennial herb is ginger (*Zingiber officinale*), which belongs to the family Zingiberaceae. Its rhizomes or roots are an efficient edible part with multiple bioactive constituents (Lai et al. 2022). They contain fibers, lecithin, proteins, fatty acids, vitamins, and volatile components including oleoresin, zingiberene, terpenes, zingerone, and zingiberol. This plant is widely used for therapeutic purposes due to its antioxidant, immunostimulant, anti-inflammatory, and antimicrobial properties (Nile and Park 2015; Mao et al. 2019).

In aquaculture, the majority of investigations have assessed feed additives under appropriate environments for fish culturing. For instance, prior research looked into single integration of carob or ginger in fish diets. They showed multiple benefits involving an improvement in immunological responses, growth, and tolerance to many pathogens and pollutants in *O. niloticus* (Yilmaz 2020; Abdelmagid et al. 2023), common carp (*Cyprinus carpio*) (Mohammadi et al. 2020), and zebrafish (*Danio rerio*) (Ahmadifar et al. 2019). On the other hand, inappropriate rearing conditions (like unchanged water or stocking density) negatively influenced fish health and immunity (Ayyat et al. 2022; Kord et al. 2022; Abdel-Aziz et al. 2024). However, data on the protective effect of *C. siliqua* combined with *Z. officinale* against un-renewed water-causing stress in fish is currently lacking. Thus, the purpose of this work was to assess the possible interactive impact of *C. siliqua* syrup and *Z. officinale* powder on immune-antioxidant response, growth rate, stress/pro-inflammatory cytokine expressions, and resistance to ammonia stress in *O. niloticus* cultivated in unchanged water.

## Materials and methods

### Ethical declaration and diet preparation

All research producers in this study were approved by the Institutional Animal Care and Use Committee of Agricultural Research Center (ARC-AMFFAU- 7–25) Egypt. *C. siliqua* syrup (CSS; Brand: Vitrac) and *Z. officinale* powder (ZOP) were purchased from the herbalist market in Arish, North Sinai, Egypt. Six tested diets (isonitrogenous and isocaloric) were prepared to meet the optimal nutritional needs of *O. niloticus* according to NRC (2011). A basal diet was used without any additives as a control (Table 1). The diets were provided with CSS (1.25%) and/or ZOP (0.5 and 1%), according to Yilmaz (2020) and Ahmed et al. (2023), respectively, which are referred to as CSS1.25, ZOP0.5, ZOP1, CSS1.25 + ZOP0.5, and CSS1.25 + ZOP1 diets. All ingredients of the basal diet were milled and mixed well with the doses of the tested additives according to the experimental protocol. After drying in an oven at 40 °C, the pellets were refrigerated.

**Table 1:** Ingredients and chemical composition of the basal diet

Ingredients	Formulation (g/kg)
Fish meal (62.2%)	120
Soybean meal (46%)	345
Corn gluten meal	40
Wheat middling	50
Wheat bran	98
Yellow corn	267.5
Limestone	4
Common salt	2
Choline chloride	0.5
Plant oils	19.5
Wheat flour	50
Vitamin & mineral^*^	3
Vitamin C	0.5
Total	1000
Chemical composition (on dry matter basis)	**%**
Crude protein	29.99
Ether extract	6.65
Ash	4.34
Crude fiber	3.99
Calcium	1.14
Lysine	1.73
Methionine	0.57
Gross energy (kJ/kg)	16,845. 40

^*^Premix (vitamins and minerals), each 1 kg feeds contains vitamin D = 1400 IU, vitamin A = 7000 IU, vitamin K_3_ = 3 mg, vitamin E = 10 mg, vitamin B_2_ = 4 mg, vitamin B_12_ = 0.01 mg, vitamin B_1_ = 1 mg, niacin = 20 mg, vitamin B_6_ = 1 mg, folic acid = 1 mg, biotin = 0.025, pantothenic acid = 8 mg, cobalt = 0.01 mg, iron = 15 mg, copper = 10 mg, selenium = 0.01 mg, iodine = 0.05 mg, zinc = 40 mg, manganese = 40 mg

#### Fish raising and experimental approach

The present experiment was conducted in the Aquaculture Unit in Desert Regions (AUDR), Faculty of Aquaculture and Marine Fisheries, Arish University, Arish, North Sinai, Egypt. Apparently, healthy fingerlings of *O. niloticus* were selected from the stock pond in AUDR with an average initial body weight (IBW) of 25.85 ± 1.42 g. They were kept for acclimation for 15 days prior to the trial. Throughout this duration, fish were hand-fed the basal ration (thrice daily at 9:00, 13:00, and 17:00).

After the acclimation time and for 6 weeks, the fish were randomly housed into six groups in 18 polyethylene cages (ten fish/cage; three replicates/group) and fed on the tested diets. The cages were well-aerated using an air blower (2 HP) via air stones with a water volume of 80 L/cage. The groups were control, CSS1.25, ZOP0.5, ZOP1, CSS1.25 + ZOP0.5, and CSS1.25 + ZOP1. Fish were reared under zero water exchange during the trial. The clinical observation of the fish was performed to record any mortality or clinical signs during the experimental period. The fish were handily fed until satiety thrice daily (9:00, 13:00, and 17:00). A siphon was applied to remove uneaten feed and fish waste from the pond bottom.

#### Water physiochemical assays

Water samples were collected from fish cages, and the physiochemical parameters were measured as water temperature, pH, and dissolved oxygen (DO) concentration every day using a multi-parameter water quality analyzer (MULP- 8 C). Total ammonia nitrogen and nitrite levels were measured every week using chemical methods (APHA 2005).

#### Growth variables and survival rate calculation

After 6 weeks of the feeding trial, the final body weight (FBW) was estimated. Hence, growth metrics were determined (Jayant et al. 2018) using the following formulas:

$$\text{Total weight gain }(\text{TWG},\text{ g}) =\text{FBW }-\text{ IBW}$$

$$\text{Average daily gain }(\text{ADG},\text{ g}) =\frac{\text{WG }\left(\text{g}\right)}{\text{Days}}$$

$$\mathrm{Specific}\;\mathrm{growth}\;\mathrm{rate}\;\left(\mathrm{SGR}\right),\%/\mathrm{day}=100\times\frac{\mathrm{Natural}\;\log\;\mathrm{FBW}-\mathrm{natural}\;\log\;\mathrm{IBW}}{\mathrm{Days}}$$

$$\text{Feed conversion ratio }(\text{FCR})= \frac{\text{Feed intake }(\text{g}/\text{fish}) }{\text{TWG }(\text{g})}$$

$$\mathrm{Survival}\;\mathrm{rate}\left(\mathrm{SR},\%\right)=100\times\frac{\mathrm{Final}\;\mathrm{fish}\;\mathrm{number}}{\mathrm{Initial}\;\mathrm{fish}\;\mathrm{number}}$$

#### Sampling

At the end of the trial period (6 weeks), three fish from each replicate were anesthetized with clove oil at a dose of 50 µg/L (El-Dakar et al. 2021) to withdraw blood from caudal blood vessels using 1-mL syringes without anticoagulant. The collected blood was centrifuged at 1750 × *g* for 15 min to separate serum for biochemical, immunological, and oxidant/antioxidant assays. Moreover, fish were euthanized by decapitation, and the liver and gills were removed. Liver samples (nine/group) were divided into two parts groups. One of them was gathered on 1 mL of QIAzol (Qiagen, Germany) and immediately frozen at 80 °C for analyzing gene expression. The other part with gill tissues was preserved in buffered neutral formalin (10%) for histological study.

#### Biochemical assays

Biochemical assessments of serum levels as alanine aminotransferase (ALT, catalog No.:292–007), aspartate aminotransferase (AST, catalog no.: 291–007), urea (catalog no.: 319–001), and glucose (catalog no.: 250–007) were calorimetrically performed (Chaney and Marbach 1962; Bergmeyer and Bernt 1974; Tietz et al. 1992) using bioscience kits of the Egyptian Corporation for Biotechnology (Cairo, Egypt). In addition, the quantity of serum cortisol was spectrophotometry determined using the ELISA method from a commercial kit (DRG Cortisol ELISA EIA‒1887, Germany) as described by Abd El-Galil et al. (2024).

#### Immunological and oxidant/antioxidant assays

The ELISA (enzyme-linked immunosorbent assay; Lambda EZ201; PerkinElmer) technique was used to detect the serum’s levels of immune globulin M (IgM; catalog no.: CSB-E12045 Fh) based on the instructions included in the kits. Lysozyme activity was turbidimetrically assessed using a suspension of *Micrococcus lysodeikticus* (Sigma-Aldrich, USA) following the prior protocol (Ellis 1990). The levels of malonaldehyde (MDA; catalog no.: MD2529), superoxide dismutase (SOD; catalog no.: SD2521), catalase (CAT; catalog no.: CA2517), and reduced glutathione content (GSH; catalog no.: GR2511) were estimated using colorimetric techniques using Bio-diagnostic reagent kits (Cairo, Egypt) (Beutler et al. 1963; Nishikimi et al. 1972; Ohkawa et al. 1979; Aebi 1984).

#### Histopathological analysis

Liver and gill specimens were taken, fixed for 24 h in 10% buffered neutral formalin solution (10%), dehydrated in progressively increasing ethanol (70–100%), cleaned in xylene, and placed in paraffin. A microtome of Leica RM 2155 (England) was used to slice paraffin slices that were 5-µm thick. Following preparation, the sections were regularly stained with hematoxylin and eosin stains and seen under a microscope following the Suvarna et al. (2018) approach.

#### Gene expression study

As directed by the supplier, QIAzol (Qiagen, Germany) was used to extract total RNA from liver tissues (50 mg) after a 6-week trial. Employing a NanoDrop® ND- 1000 UV–Vis spectrophotometer (Thermo Scientific, Massachusetts, United States) and at 260/280 wavelengths, the isolated RNA was measured. A reverse transcriptase kit from Applied Bio-system (California, USA) was used to generate the complementary DNA in line with the manufacturer’s directions. The primers used were supplied by Sangon Biotech (Beijing, China) and listed in Table 2. The real-time heat cycler Rotor-Gene Q 2 plex (Qiagen, Germany) was used to perform the quantitative polymerase chain reaction consistent with the procedure of Ibrahim et al. (2024). Fold changes were computed using the 2^−ΔΔCT^ method that was previously detailed (Livak and Schmittgen 2001). The expression of antioxidant (*SOD*, *CAT*, and glutathione peroxidase (*GPx*)), stress (heat shock protein 70 (*HSP70*)), and pro-inflammatory cytokines (interleukin- 1β (*IL- 1β*) and tumor necrosis factor-alpha (*TNF-α*))-related-genes were evaluated. The beta-actin (*β-actin*) gene was employed as a housekeeping gene.

**Table 2:** Primers sequences for gene expression assay

Target gene	Primer sequence	Accession number	Primer efficiency
Beta actin	CAGGATGCAGAAGGAGATCACA CGATCCAGACGGAGTATTTACG	XM_003443127.5	96.42
Superoxide dismutase	TCACAGCAAGCACCATGCTA GCAACCTGTGTTGTCACGTC	XM_003449940.5	100.25
Catalase	TCCTGAATGAGGAGGAGCGA AAACGTGCAAAGTGGCATCC	JF801726.1	99.25
Glutathione peroxidase	GTGCCCTGCAATCAGTTTGG CGAGGAGCTGGAACTTTGGT	NM_001279711.1	97.62
Heat shock protein 70	TCACCACCTACTCCGACAAC CCACCGCAGACACATTCAAA	FJ207463.1	94.56
Interleukin- 1β	CTCATGTCTGTCCGCTACCC TGAAGCTTCTGTAGCGTGGG	KF747686.1	98.36
Tumor necrosis factor	CTGCTCCCTTCCACTCCTTG CCGCTATCTGTGAGAGGCTG	XM_013266975.3	94.65

####  Stress assay (ammonia toxicity)

At the end of 6 weeks, the remaining fish in each treatment were subjected to 0.54 g/L ammonium chloride, ACS (ClH_4_N; percentage assay, 99.5%; molecular weight, 53.49 g/mol;Thermo Scientific Chemicals, India), for 96 h (Yilmaz 2020). To achieve this, a stock solution of ammonium chloride was prepared and added to the cages 12 h apart. The ammonia content in the cages was measured colorimetrically (Tan et al. 2018), and fish mortalities were tallied every day.

#### Data analysis

Bartlett and Kolmogorov–Smirnov assays were used to verify the normality and homogeneity of data. After that, data were analyzed by one-way analysis of variance (ANOVA) followed by Duncan’s multiple comparison test to assess the mean variances. Results were considered significant at *P* < 0.05. The software program of SPSS version 18.0 (SPSS Inc., Chicago, USA) was used for the analysis.

## Results

### Water physiochemical assays

Table 3 shows no obvious differences in water temperature and pH between treatments. There was a marked increase in the DO level and a decline in the total ammonia nitrogen and nitrite values in the group fed CSS and/or ZOP-enriched diets relative to the control (*P* < 0.05).

**Table 3:** Water quality barometers of Nile tilapia reared without water renewal and fed *C. siliqua* syrup (CSS) and/or *Z. offcinale* powder (ZOP)-fortified diets for 6 weeks

Groups	Temperature (°C)	pH	Dissolved oxygen (mg/L)	Total ammonia nitrogen (mg/L)	Nitrite (mg/L)
Control	24.10	8.76	4.80^b^	1.34^a^	1.40^a^
CSS1.25	24.00	8.48	5.90^a^	0.81^b^	1.03^b^
ZOP0.5	23.90	8.52	5.95^a^	0.83^b^	1.24^b^
ZOP1	24.00	8.55	5.95^a^	0.88^b^	1.18^b^
CSS1.25 + ZOP0.5	23.85	8.47	5.85^a^	0.85^b^	1.12^b^
CSS1.25 + ZOP1	24.22	8.40	5.60^a^	0.87^b^	1.20^ab^
*P*-value	0.52	0.83	0.04	0.04	0.002
SEM^*^	0.06	0.05	0.39	0.09	0.07

Control: fish-fed basal diet without additives. CSS1.25, ZOP0.5, ZOP1, CSS1.25 + ZOP0.5, and CSS1.25 + ZOP1: fish-fed basal diets supplemented with CSS (1.25%) and/or ZOP (0.5 and 1%). Means with different superscripts in the same column are significantly different at *P* < 0.05

^*^SEM standard error means

### Growth variables and survival

Table 4 shows a decline in the FBW, TWG, and SGR and an elevation in the FCR by CSS1.25 and CSS1.25 + ZOP1 diets compared to the control. Fish of the ZOP1 group had the highest growth metrics and the lowest FCR values (*P* < 0.05) followed by ZOP0.5 and CSS1.25 + ZOP0.5 groups. In the same line, ZOP0.5, ZOP1, and CSS1.25 + ZOP0.5 groups recorded the highest SR (100%), meanwhile, the CSS1.25 group showed the lowest one (85%).

**Table 4:** Growth barometers and survival of Nile tilapia reared without water renewal and fed *C. siliqua* syrup (CSS) and/or *Z. offcinale* powder (ZOP)-fortified diets for 6 weeks

Groups	IBW (g)	FBW (g)	TWG (g)	ADG (g)	SGR (%/day)	FCR	Survival (%)
Control	25.62	52.01^bc^	26.39^bc^	0.63^c^	1.68^bc^	1.57^b^	87.50
CSS1.25	25.75	47.25^c^	21.50^c^	0.51^c^	1.44^c^	2.58^a^	85.00
ZOP0.5	26.00	58.18^ab^	32.18^ab^	0.76^b^	1.91^ab^	1.65^b^	100
ZOP1	26.00	64.19^a^	38.19^a^	0.91^a^	2.14^a^	0.89^c^	100
CSS1.25 + ZOP0.5	26.12	57.28^ab^	31.16^ab^	0.74^b^	1.86^ab^	1.58^b^	100
CSS1.25 + ZOP1	25.62	48.53^c^	22.91^c^	0.54^c^	1.51^c^	1.82^b^	87.50
*P*-value	0.99	0.03	0.01	0.01	0.004	< 0.001	-
SEM^*^	1.42	3.08	3.07	0.07	0.13	0.13	-

Control: fish-fed basal diet without additives. CSS1.25, ZOP0.5, ZOP1, CSS1.25 + ZOP0.5, and CSS1.25 + ZOP1: fish-fed basal diets supplemented with CSS (1.25%) and/or ZOP (0.5 and 1%). Means with different superscripts in the same column are significantly different at *P* < 0.05

^*^SEM standard error means, *IBW* initial body weight, *FBW* final body weight, *TWG* total weight gain, *ADG* average daily gain, *SGR* specific growth rate, *FCR* feed conversion ratio

### Biochemical assays

Table 5 reveals the values of serum biochemical indices (ALT, AST, urea, glucose, and cortisol), where CSS and/or ZOP diets considerably declined (*P* < 0.05) these variables relative to the control except CSS1.25 + ZOP1 diet for ALT, AST, and glucose values. In addition, the ZOP1 group had the lowest levels of glucose and cortisol.

**Table 5:** Biochemical barometers of Nile tilapia reared without water renewal and fed *C. siliqua* syrup (CSS) and/or *Z. offcinale* powder (ZOP)-fortified diets for 6 weeks

Groups	ALT (U/L)	AST (U/L)	Urea (mg/dL)	Glucose (mg/dL)	Cortisol (ng/mL)
Control	36.60^a^	73.80^a^	55.90^a^	118.00^a^	28.64^a^
CSS1.25	27.00^b^	50.50^b^	14.30^b^	115.00^ab^	19.22^b^
ZOP0.5	26.00^b^	44.30^c^	14.70^b^	102.00^c^	19.54^b^
ZOP1	27.00^b^	48.8^c^	12.70^b^	101.00^c^	14.51^c^
CSS1.25 + ZOP0.5	26.75^b^	42.7^c^	10.01^b^	108.00^c^	18.27^b^
CSS1.25 + ZOP1	35.40^a^	67.40^ab^	12.75^b^	117.00^ab^	19.94^b^
*P*-value	0.02	< 0.001	< 0.001	0.01	0.02
SEM^*^	19.18	5.85	3.47	5.88	3.13

Control: fish-fed basal diet without additives. CSS1.25, ZOP0.5, ZOP1, CSS1.25 + ZOP0.5, and CSS1.25 + ZOP1: fish-fed basal diets supplemented with CSS (1.25%) and/or ZOP (0.5 and 1%). Means with different superscripts in the same column are significantly different at *P* < 0.05

^*^SEM standard error means, *ALT* alanine aminotransferase, *AST* aspartate aminotransferase

### Immunological and oxidant/antioxidant assays

Table 6 shows a substantial increase (*P* < 0.05) in the IgM level and lysozyme activity by CSS and/or ZOP diets over the control except for the CSS1.25 + ZOP1 diet. Dietary CSS and/or ZOP significantly decreased MDA and boosted SOD and GSH levels except for the CSS1.25 diet for GSH (*P* < 0.05) as seen in Table 6. In contrast, CAT activity did not alter by CSS and/or ZOP diets. Dietary ZOP1 and CSS1.25 + ZOP0.5 diets significantly exhibited the best values (*P* < 0.05).

**Table 6:** Immunological and oxidant/antioxidant responses of Nile tilapia reared without water renewal and fed *C. siliqua* syrup (CSS) and/or *Z. offcinale* powder (ZOP)-fortified diets for 6 weeks

Groups	IgM (µg/mL)	Lysozyme(U/mL)	MDA (nmol/L)	SOD (U/L)	CAT (U/L)	GSH (mg/L)
Control	5.60^c^	67.00^c^	245.00^a^	57.60^c^	23.40	6.50^c^
CSS1.25	6.35^b^	81.01^ab^	122.00^c^	64.30^b^	22.60	5.30^c^
ZOP0.5	6.40^b^	79.00^ab^	112.50^c^	64.35^b^	25.70	9.70^b^
ZOP1	13.30^a^	95.03^a^	109.00^c^	86.40^a^	23.22	18.45^a^
CSS1.25 + ZOP0.5	12.10^a^	89.80^a^	117.50^c^	89.70^a^	26.07	19.60^a^
CSS1.25 + ZOP1	3.50^c^	77.00^bc^	173.00^b^	85.02^a^	24.30	11.30^b^
*P*-value	< 0.001	0.01	< 0.001	0.001	0.94	0.003
SEM^*^	0.84	4.36	11.05	4.71	4.15	2.22

Control: fish-fed basal diet without additives. CSS1.25, ZOP0.5, ZOP1, CSS1.25 + ZOP0.5, and CSS1.25 + ZOP1: fish-fed basal diets supplemented with CSS (1.25%) and/or ZOP (0.5 and 1%). Means with different superscripts in the same column are significantly different at *P* < 0.05

^*^SEM standard error means, *IgM* immunoglobulin M, *MDA* malondialdehyde, *SOD* superoxide dismutase, *CAT* catalase, *GSH* reduced glutathione content

### Histopathological findings

**Fig. 1:**
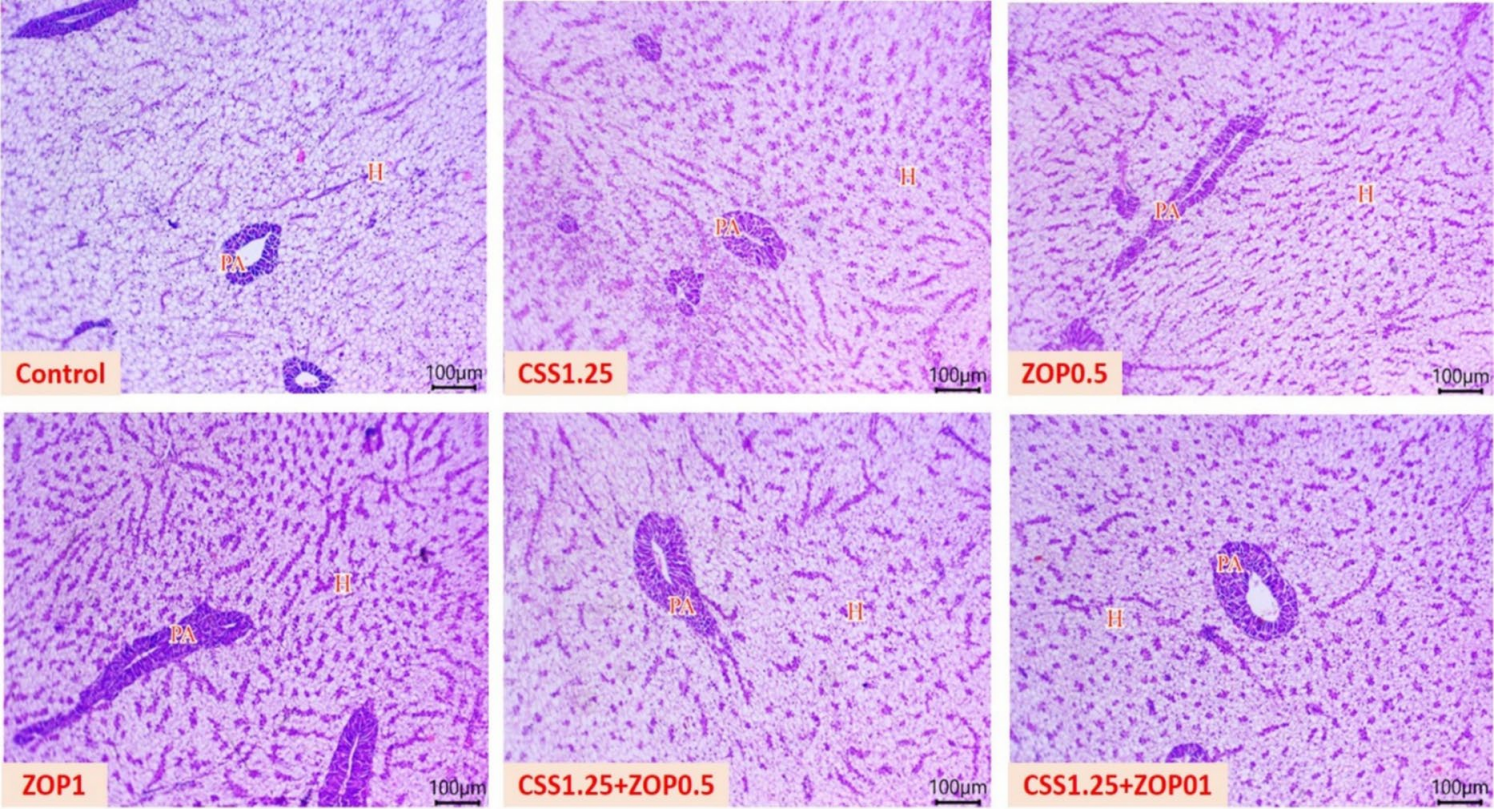
Photomicrograph of hepato-pancreas tissue sections (H&E; scale bar, 100 µm) of Nile tilapia reared without water renewal and fed *C. siliqua* syrup (CSS) and/or *Z. offcinale* powder (ZOP)-fortified diets for 6 weeks showing preserved morphological structures of vacuolated hepatocytes, pancreatic acinar epithelium around branches of the portal vein, and gradual increase in the activity of apical granules within pancreatic acinar epithelium in all treated groups. Hepatocytes (H) and exocrine pancreatic acini (PA). Control: fish-fed basal diet without additives. CSS1.25, ZOP0.5, ZOP1, CSS1.25 + ZOP0.5, and CSS1.25 + ZOP1: fish-fed basal diets supplemented with CSS (1.25%) and/or ZOP (0.5 and 1%)

**Fig. 2:**
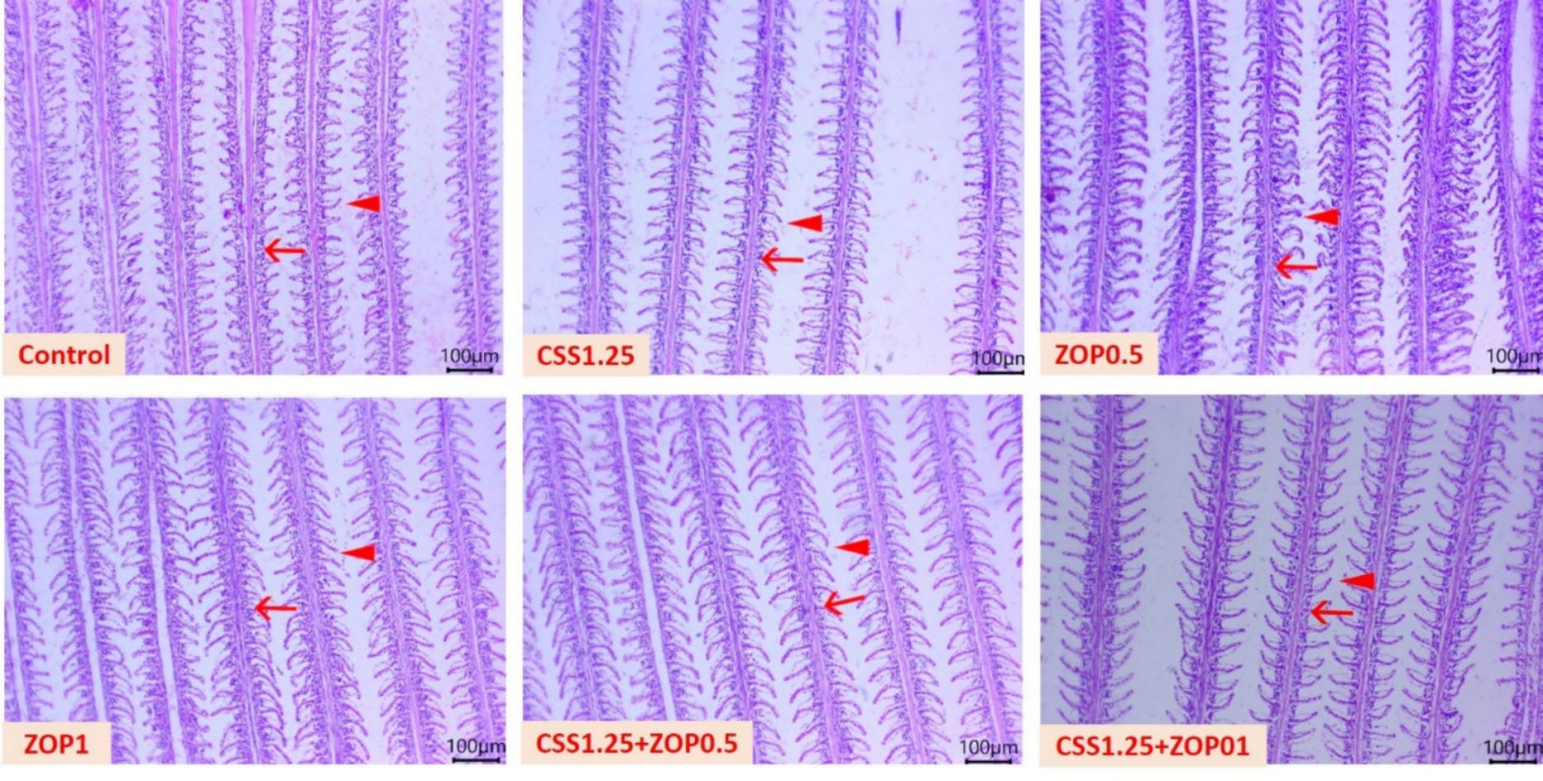
Photomicrograph of gill tissue sections (H&E; scale bar, 100 µm) of Nile tilapia reared without water renewal and fed *C. siliqua* syrup (CSS) and/or *Z. offcinale* powder (ZOP)-fortified diets for 6 weeks showing normal histological architectures of the primary gill filaments (arrows), secondary gill filaments (arrowheads), and other stromal tissues, besides a gradual improvement in the arrangement of gill filaments in all treated groups. Control: fish-fed basal diet without additives. CSS1.25, ZOP0.5, ZOP1, CSS1.25 + ZOP0.5, and CSS1.25 + ZOP1: fish-fed basal diets supplemented with CSS (1.25%) and/or ZOP (0.5 and 1%)

Figure 1 demonstrates histological outcomes of hepato-pancreas tissues, where preserved morphological structures of vacuolated hepatocytes, and pancreatic acinar epithelium around branches of the portal vein were obvious in all treatments. In addition, a gradual increase in the activity of apical granules within pancreatic acinar epithelium was seen. Moreover, gill tissues (Fig. 2) showed non-observable pathological lesions with normal histological architectures of epithelial lining, primary and secondary gill filaments, and other stromal tissues. In addition, gradual improvement in the arrangement of gill filaments was observed in all treatments.

### Expression of antioxidant, stress, and pro-inflammatory cytokines

**Fig. 3:**
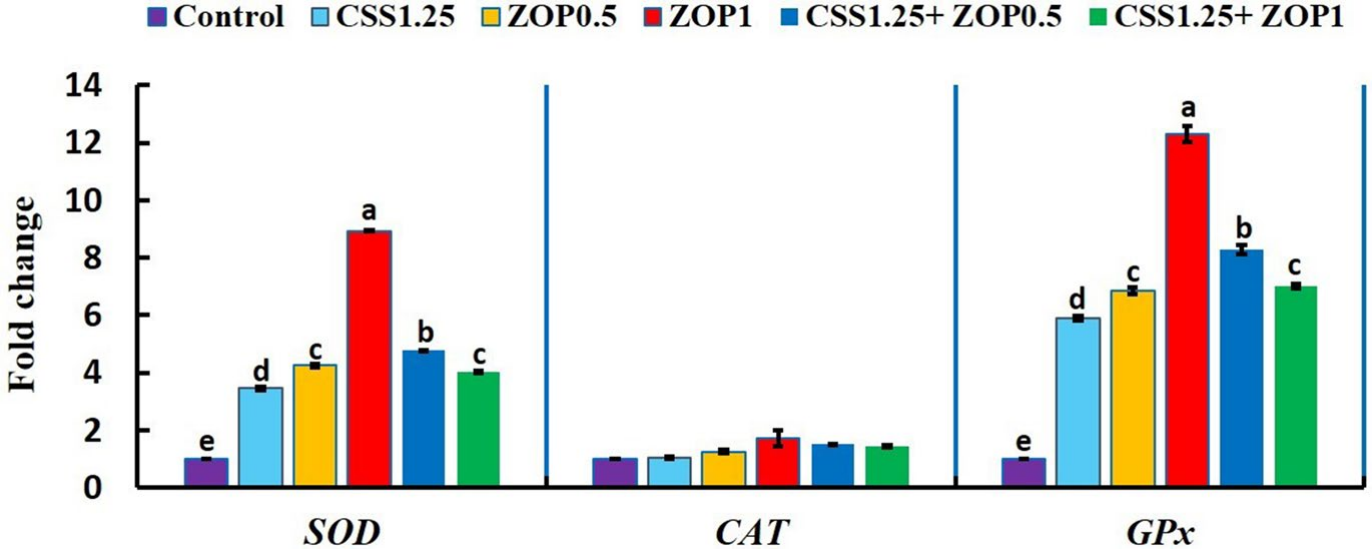
Expression of hepatic antioxidant-associated genes (*SOD*, *CAT*, and *GPx*) of Nile tilapia reared without water renewal and fed *C. siliqua* syrup (CSS) and/or *Z. offcinale* powder (ZOP)-fortified diets for 6 weeks. Control: fish-fed basal diet without additives. CSS1.25, ZOP0.5, ZOP1, CSS1.25 + ZOP0.5, and CSS1.25 + ZOP1: fish-fed basal diets supplemented with CSS (1.25%) and/or ZOP (0.5 and 1%). Bars with distinct superscripts showed substantial variations (*P* < 0.05). *SOD*, superoxide dismutase; *CAT*, catalase; *GPx*, glutathione peroxidase

Figure 3 demonstrates that CSS and/or ZOP diets substantially upregulated (*P* < 0.05) the *SOD* and *GPx* gene expressions relative to the control. The ZOP1 diet recorded the highest expression. The fold change was 3.45-, 4.25-, 8.93-, 4.77-, and 4.02-fold for *SOD* and 5.89-, 6.85, 12.29-, 8.28-, and 7.00-fold for *GPx* in the CSS1.25, ZOP0.5, ZOP1, CSS1.25 + ZOP0.5, and CSS1.25 + ZOP1 groups, respectively. However, the expression of the *CAT* gene did not change between treatments.

**Fig. 4:**
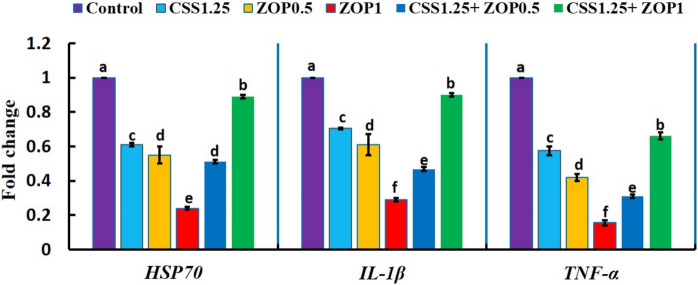
Expression of hepatic stress (*HSP70*) and pro-inflammatory cytokines (*IL- 1β* and *TNF-α*) inflammation-associated genes of Nile tilapia reared without water renewal and fed *C. siliqua* syrup (CSS) and/or *Z. offcinale* powder (ZOP)-fortified diets for 6 weeks. Control: fish-fed basal diet without additives. CSS1.25, ZOP0.5, ZOP1, CSS1.25 + ZOP0.5, and CSS1.25 + ZOP1: fish-fed basal diets supplemented with CSS (1.25%) and/or ZOP (0.5 and 1%). Bars with distinct superscripts showed substantial variations (*P* < 0.05). *HSP70*, heat shock protein 70; *IL- 1β*, interleukin- 1β; *TNF-α*, tumor necrosis factor-alpha

Figure 4 exhibited that the expression of *HSP70*, *IL- 1β*, and *TNF-α* was downregulated (*P* < 0.05) by the CSS and/or ZOP diets, where the lowest expression level was in the ZOP1 diet. The fold change was 0.61-, 0.55-, 0.24-, 0.51-, and 0.89-fold for *HSP70*; 0.70-, 0.61-, 0.29-, 0.46-, and 9.00-fold for *IL- 1β*; and 0.57-, 0.42-, 0.15-, 0.31-, and 0.66-fold for *TNF-α* in the CSS1.25, ZOP0.5, ZOP1, CSS1.25 + ZOP0.5, and CSS1.25 + ZOP1 groups, respectively.

### Resistance to ammonia toxicity

**Fig. 5:**
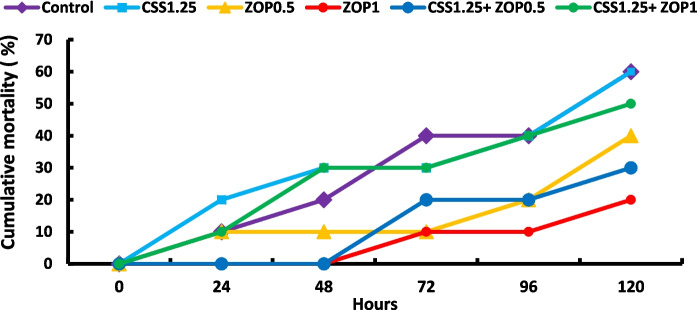
Cumulative mortality of Nile tilapia reared without water renewal and fed *C. siliqua* syrup (CSS) and/or *Z. offcinale* powder (ZOP)-fortified diets for 6 weeks and after that, fish were exposed to ammonia toxicity. Control: fish-fed basal diet without additives. CSS1.25, ZOP0.5, ZOP1, CSS1.25 + ZOP0.5, and CSS1.25 + ZOP1: fish-fed basal diets supplemented with CSS (1.25%) and/or ZOP (0.5 and 1%)

Figure 5 depicts the cumulative mortality of fish exposed to ammonia toxicity, where it was as follows: 60, 60, 40, 20, 30, and 50% in the control, CSS1.25, ZOP0.5, ZOP1, CSS1.25 + ZOP0.5, and CSS1.25 + ZOP1 groups, respectively.

## Discussion

Fish production depends greatly on water quality, which also has an immense impact on fish growth, health, and maintenance of an ideal aquatic ecosystem (Verma et al. 2022). In our investigation, total ammonia nitrogen and nitrite decreased, while DO levels rose in CSS and/or ZOP-fed groups. These results can be brought about by the significant reduction in the release of nitrogen molecules that are expelled as a result of enhanced feed digestion and uptake of nutrients (Fabay et al. 2022). Dietary CSS and ZOP considerably improved water quality by a decrease in the outflow of nitrogen molecules (total ammonia nitrogen and nitrite). The conserved histological picture of the gills in our findings supported these influences. Previous research showed that *C. siliqua* kibbles enhanced the quality of wastewater (Díaz et al. 2023). In addition, feeding ZOP to *O. niloticus* had a beneficial impact on water quality with no histological alteration on gills (Mohammed et al. 2020).

Growth as a biological function is affected by genetic, physiological, environmental, and dietary aspects (Akalu 2021). In this work, dietary ZOP alone or combined with CSS (CSS1.25 + ZOP0.5 group) improved growth metrics and feed utilization (FCR), as well as fish survival. The ZOP1 group had the highest results. The rationale for dietary ZOP’s positive impacts is proteinase, which is abundantly found in ZOP, aids in the digestion and absorption of amino acids (Hashim et al. 2011). ZOP has a beneficial effect on gut bacterial flora and helps to obtain more nutrients. In addition, ZOP possesses antibacterial properties that can eradicate harmful microbes in the gut and can alleviate intestinal distress (Lai et al. 2022). The terpene and zingiberene included in ZOP are well-known for their flavor and appealing scent. In addition, gingerol of ZOP, can improve the feed’s palatability and promote effective feed absorption in the gut (¨Ozcan 2022; Gao et al. 2022). Moreover, the high growth accounted for by ZOP may be linked with the boosted growth hormone level and digestive enzymes by dietary ZOP (Ahmed et al. 2023). According to comparable results, dietary ZOP or its extracts promoted the growth and survival of *C. carpio* (Mohammadi et al. 2020), *O. niloticus* (Naliato et al. 2021), and black rockfish (*Sebastes schlegelii*) (Oh et al. 2022). Moreover, the growth of striped catfish (*Pangasianodon hypophthalmus*) cultured in outdoor conditions was enhanced by dietary ZOP (Ashry et al. 2023). On the contrary, the CSS1.25 diet declined the growth rate, and this outcome was verified in *O. niloticus* by Yilmaz et al. (2018) and Yilmaz (2020). This may be due to the high tannin concentration, which tends to influence digestion by interacting with digestive enzymes and proteins (Antonopoulou et al. 2022).

Since liver and kidney functions represent the entire nutritional condition and water contaminants exposure, they are regarded as fundamental diagnostic signs in aquaculture (Rohani 2023). Dietary CSS and ZOP decreased the AST, ALT, and urea values as hepato-renal markers. This result suggests better health, demonstrating CSS and ZOP’s protective effects. These influences were validated with the preserved histological integrity of the liver in our findings. The antioxidant components in CSS (-epicatechin gallate, quercetin, and (−)-epigallocatechin gallate) and ZOP (gingerols and shogaols) can act as radical scavengers and shield liver and renal cells from oxidative damage, leading to improved function and health (Maghsoudi et al. 2011; Stavrou et al. 2018; Jafarinejad et al. 2020). Also, ZOP may increase the liver’s ability to detoxify by decreasing the buildup of toxins and boosting detoxifying enzymes (Haniadka et al. 2013). The hepatic and renal-protective potentials of *C. siliqua* or ZOP were reported by Alsherbiny et al. (2019), Gabr et al. (2019), and Martić et al. (2022). Similarly, Abdelmagid et al. (2023) and Ahmed et al. (2023) noticed a drop in the hepato-renal function biomarkers of *O. niloticus*-fed diets enriched with ZOP.

Glucose and cortisol levels serve as robust physiological biomarkers for assessing fish health (Raposo de Magalhães et al. 2020). Dietary integration of CSS and ZOP declined stress markers (glucose and cortisol), which confirms their potent anti-stress impacts as reported in previous investigations (Moon et al. 2017; Ünal et al. 2023). Regarding the low glucose level by CSS, the d-pinitol of *C. siliqua*, which triggers insulin signaling leads to boosting glucose uptake. Also, it can suppress gluconeogenesis and encourage glycogen synthesis (Spizzirri et al. 2024). Polyphenols of *C. siliqua* chelate carbohydrates, fibers, and fats, hence decreasing intestinal absorption (Williamson 2013). Additionally, α-glucosidase and α-amylase enzymes, which convert carbohydrates into glucose, are reported to be inhibited by *C. siliqua* (Qasem et al. 2018). Moreover, ZOP’s high flavonoid and polyphenol content boosts glucose absorption by activation of insulin (Arablou et al. 2014). ZOP reduces inflammation and hence, it enhances peripheral tissues’ insulin signaling, insulin sensitivity, and glucose clearance—all of which are essential for the regulation of blood glucose (Jafarnejad et al. 2017). A similar outcome was reported by dietary ZOP (Fazelan et al. 2020). In addition, Apines-Amar et al. (2013) and Fazelan et al. (2020) revealed a decline in the cortisol level of brown-marbled grouper (*Epinephelus fuscoguttatus*) and *C. carpio* by ZOP diets.

Fish, like other vertebrates, have innate and adaptive immunity, which plays a critical part in safeguarding them against diseases (Mokhtar et al. 2023). One of the most crucial molecules of innate immunity, lysozyme plays a role in the breakdown of gram-positive bacteria’s peptidoglycan layer of the cell membrane (Li et al. 2021). The primary components of the adaptive immune response against infections are immunoglobulins. In fish serum, IgM is thought to be the most prevalent immunoglobulin (Mashoof and Criscitiello 2016). In this study, dietary CSS and/or ZOP enhanced IgM and lysozyme activity emphasizing a stronger immunological response. This response besides their antioxidant action could boost fish survival and decrease mortality after exposure to ammonia toxicity. The best findings were in the ZOP1 and CSS1.25 + ZOP0.5 groups. *C. siliqua* has a high content of polyphenolic chemicals with their immunostimulant action (Makris and Kefalos 2004). In the same way, Yilmaz (2020) pointed out an increased immune response (lysozyme and myeloperoxidase activity) of *O. niloticus* by dietary CSS. Prior work (Elhalim et al. 2017) reported a stimulated immune status of rats exposed to cyclophosphamide by *C. siliqua* pods. Also, the phytochemical constituents (gingerol, shogaols, paradols, oxalate, phytate, tannin, and saponin) and aromatic components found in ZOP may contribute to a strengthened immune response (Ude et al. 2018; Mao et al. 2019). Comparable outcomes (El-Sebai et al. 2018; Ude et al. 2018; Fazelan et al. 2020; Mohammadi et al. 2020; Ahmed et al. 2023) verified that dietary ZOP or its extracts notably raised the immune status of *O. niloticus*, *C. carpio*, and African catfish (*Clarias gariepinus*).

Through the maintenance of redox equilibrium and the oversight of the antioxidant system imbalances, fish antioxidant enzymes play a critical role in lowering oxidative damage. One of these enzymes, which protects cells by detoxification of superoxide radicals is SOD (Shi et al. 2022). In addition, GSH is the most prevalent low-molecular-weight thiol that functions exactly as an oxy-radical scavenger (Ogunwole et al. 2021). Here, a significant drop in MDA level and an increase in the SOD and GSH activity as well as their linked genes (*SOD* and *GPx*) by dietary CSS and ZOP. Additionally, the ZOP1 and CSS1.25 + ZOP0.5 groups performed the best. This proved a surge in antioxidant capability and a decrease in oxidative stress as well as protection of tissues from damage revealing an improved fish health. This outcome was verified by an improvement in the histological architecture of the liver and gills in our results. CSS’s high antioxidant activity over reference antioxidants stems from its abundance of polyphenols (Cegledi et al. 2024). Dietary *C. siliqua* could upregulate *SOD2* gene expression, hence boosting antioxidant defenses (Pelegrin-Valls et al. 2023). Moreover, ZOP is rich in polyphenols, flavonoids, saponins, and tannins, all of which have potent antioxidant traits as reported by Zhang et al. (2022). Shogaols, paradols, gingerol, and diarylheptanoids as polyphenolic molecules are present in high concentration in ZOP and exert strong antioxidant potential as reported by Mao et al. (2019). These findings have been validated by earlier investigations in *O. niloticus*, *C. carpio*, and Rohu (*Labeo rohita*) (Fazelan et al. 2020; Yilmaz 2020; Rawat et al. 2022; Abdelmagid et al. 2023).

Looking at gene expression in fish is crucial for understanding their inflammatory and immune-antioxidant status. *HSP70* is crucial for a variety of physiological functions and for protecting against environmental stresses (Mengal et al. 2023; Abdel Rahman et al. 2025a, b). Two pro-inflammatory cytokines that are produced when an inflammatory state exists are *IL- 1β* and *TNF-α* (Zou and Secombes 2016). In this work, dietary ZOP and/or CSS significantly downregulated *HSP70*, *IL- 1β*, and *TNF-α* genes suggesting strong anti-inflammatory effectiveness and immunological response. The α-linolenic acid as a major constituent of *C. siliqua* was recognized to exert anti-inflammatory activity (Otuechere and Farombi 2020; Atta et al. 2023). Linolenic acid reduced the expression of pro-inflammatory cytokines in vitro (Morin et al. 2022). Also, the extract of *C. siliqua* exerted anti-inflammatory potential in doxorubicin-exposed rats by downregulation of pro-inflammatory cytokines (*IL- 1β* and *TNF-α*) (Atta et al. 2023). Also, this finding could be explained by the phytoconstituents of ZOP (8-shoagol, zingerone, and 6-shoagol) by their anti-inflammatory characteristic (Blaxhall and Daisley 1973). In addition, ZOP downregulates the nuclear factor-kappa β gene, which in turn lowers the expression of pro-inflammatory cytokines, hence hindering inflammation (Wang et al. 2017). A prior study (Zhang et al. 2016) showed that *Z. officinale* nano-form possesses a strong anti-inflammatory effect by lowering the levels of pro-inflammatory cytokines and increasing anti-inflammatory cytokines.

## Conclusions

This work is to look at the nutritional benefits of dietary ZOP, either by itself or in conjunction with CSS, in *O. niloticus* raised without water change. A dose of 1% ZOP alone or a 0.5% ZOP and 1.25% CSS combination is advocated to improve physiological and immuno-antioxidant status and promote growth. Furthermore, these diets downregulated the expression of genes linked to stress and inflammation and upregulated that linked with antioxidants, as well as enhanced resistance to ammonia stress. To assess how these dietary additives affect other health markers in other fish species, further investigation is recommended.

## Data Availability

No datasets were generated or analysed during the current study.
